# Supplementary material for: Novel Magnesium- and Silver-Loaded Dressing Promotes Tissue Regeneration in Cutaneous Wounds
Source: Int J Mol Sci. 2024 Aug 28;25(17):9311. doi: 10.3390/ijms25179311 (PMC11395027; doi:10.3390/ijms25179311)
Supplement: Supplementary file 1 [file ijms-25-09311-s001.zip › ijms-3065039-supplementary.pdf]

Supplementary Information

Fig. S1

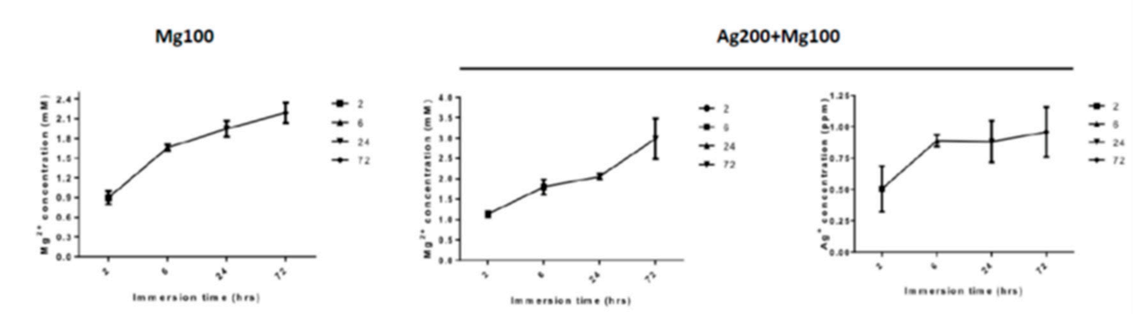

	2 hrs	6 hrs	24 hrs	72 hrs
[Mg^{2+}]	0.89 mM	1.66 mM	1.95 mM	2.19 mM

	2 hrs	6 hrs	24 hrs	72 hrs
[Mg^{2+}]	1.13 mM	1.8 mM	2.06 mM	2.98 mM
[Ag^{+}]	0.5 ppm	0.89 ppm	0.88 ppm	0.96 ppm

Fig. S1. Released property of Mg100 and Ag200+Mg100 dressings.

Mg100 and Ag200+Mg100 dressings exhibited a stable release pattern of Mg and Ag ions in a time dependent manner. (left: Mg concentration in Mg 100, middle: Mg concentration in Ag200+Mg100, right: Ag concentration in Ag200+Mg100)

Fig. S2

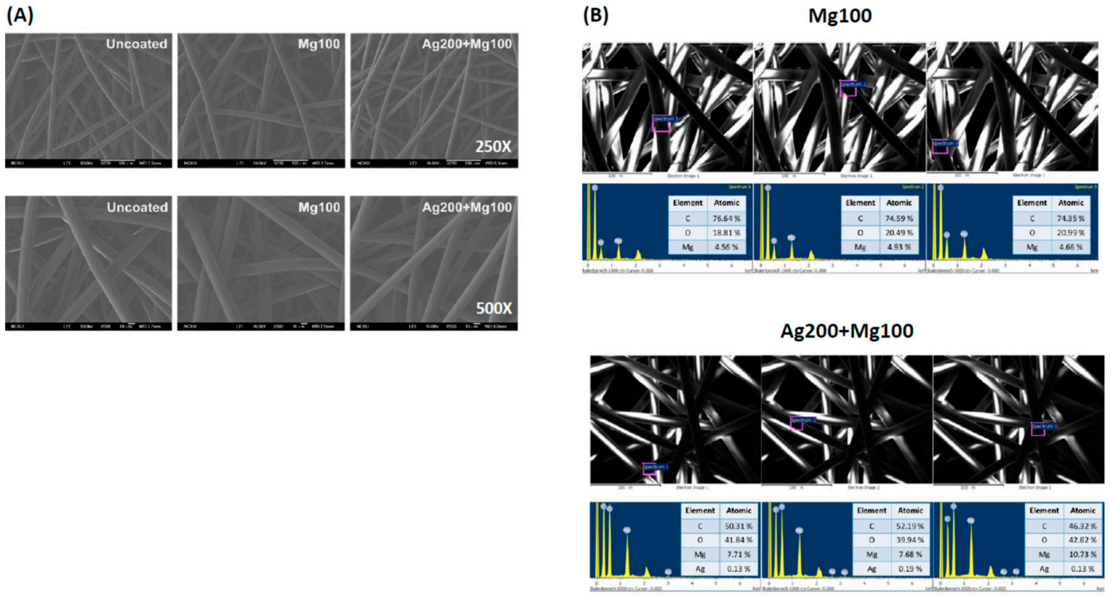

Fig. S2. Scanning electron microscopy micrographs of Mg100 and Ag200+Mg100

dressings

(A) The surface morphology of the sputtered metal ions was observed by scanning electron microscope (SEM) at 250X and 500X power field. (B) Energy-dispersive X-ray spectroscopy (EDS) analysis of Ag and Mg in Mg100 and Ag200+Mg100 dressings.

Fig. S3

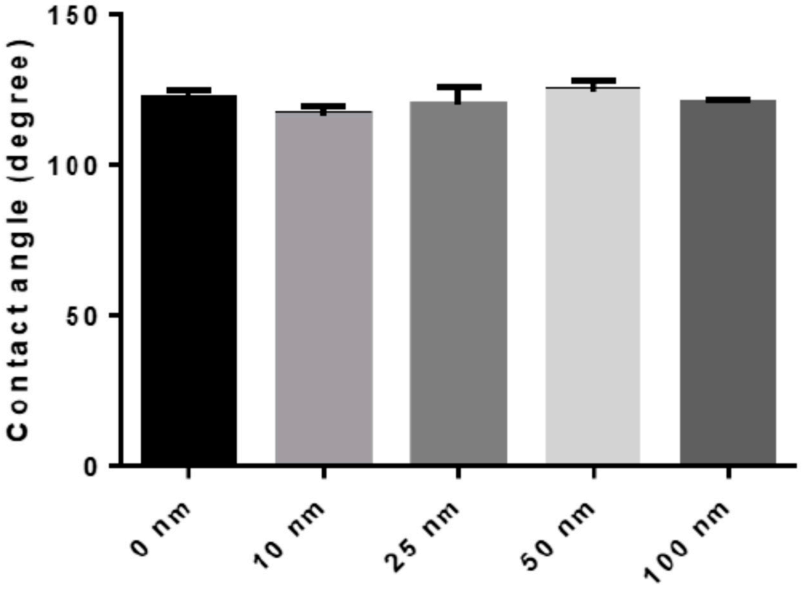

Fig. S3. Surface wettability of Mg100 and Ag200+Mg100 dressings.

The contact angle greater than 90 degrees is needed to maintain its hydrophobic property to avoid stickiness of the dressing to the tissue wound. No differences of the contact angle between uncoated dressing and all types of Mg dressings (n=3)

Fig. S4

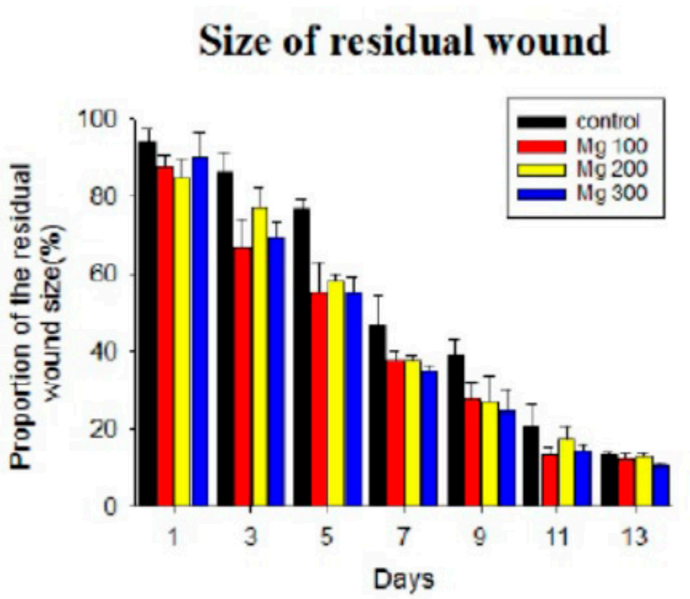

Fig. S4. Response of wound closure rate in different Mg dressings

Similar wound-closure rate is presented in 3 different Mg dressings (Mg100, Mg200 and Mg300, n=3).

Fig. S5

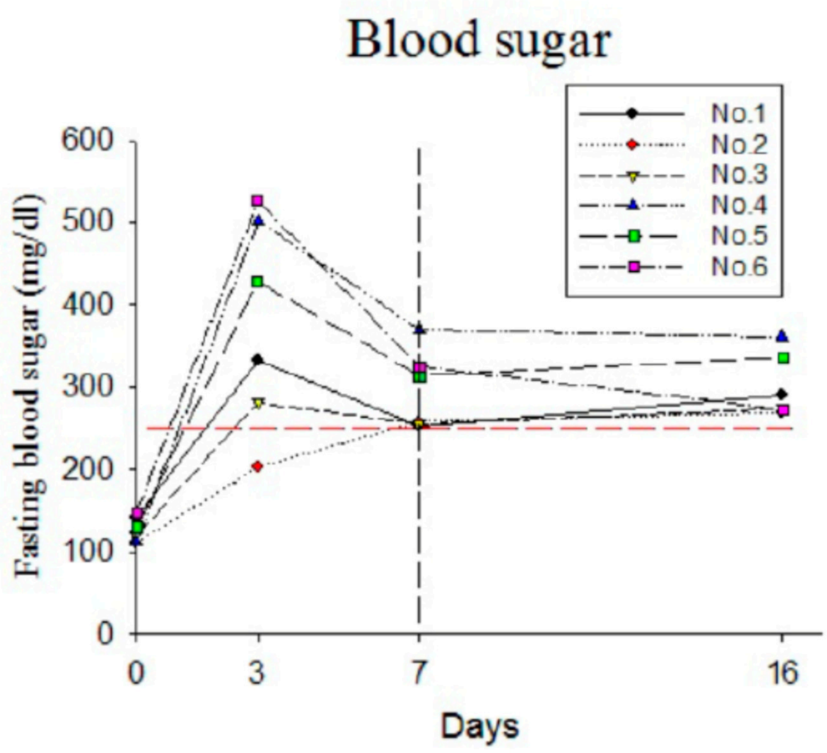

Fig. S5. Induction of diabetic rats

After 4 weeks of feeding, the rats were fasted for 3 days and injected with Streptozotocin 35 mg/kg from the tail vein. Measurement of plasma blood glucose in the fasting state was done on the 2nd and 6th days after induction. Six diabetic rats were successfully induced when fasting blood glucose was higher than 250 mg/dl.

Fig. S6

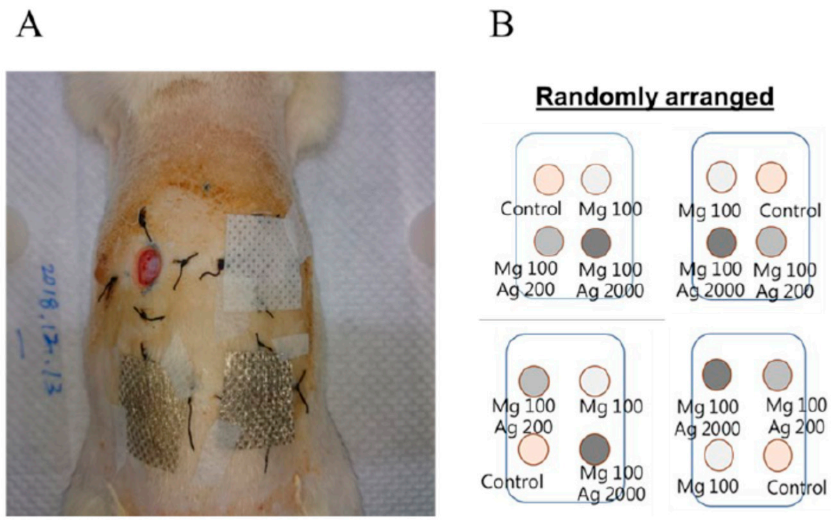

Fig. S6. Excisional wound healing model of the mice

Four full-thickness excisional wounds were made on the dorsum of the mice using a diameter of 10-mm biopsy punch. Wound edge was affixed with 4-0 nylon for 4 stitches. Four wounds were randomly covered with uncoated polyethylene, Mg100, Ag200+Mg100, Ag2000+Mg100 dressings.
